# How Vaccine Ambivalence Can Lead People Who Inject Drugs to Decline COVID-19 Vaccination and Ways This Can Be Addressed: Qualitative Study

**DOI:** 10.2196/35066

**Published:** 2022-03-23

**Authors:** Ian David Aronson, Alex S Bennett, Mary-Andrée Ardouin-Guerrier, German Rivera-Castellar, Brent Gibson, Samantha Santoscoy, Brittney Vargas-Estrella

**Affiliations:** 1 Department of Social and Behavioral Sciences School of Global Public Health New York University New York, NY United States; 2 New York Harm Reduction Educators New York, NY United States

**Keywords:** SARS-CoV-2, COVID-19, people who inject drugs, vaccine, vaccine hesitancy, barrier, vaccination, drugs, hesitancy, qualitative, impact, interview, United States, communication, danger, community

## Abstract

**Background:**

People who inject drugs are disproportionately impacted by SARS-CoV-2 and COVID-19, yet they do not frequently accept vaccination against SARS-CoV-2 when offered.

**Objective:**

This study aimed to explore why people who inject drugs decline free vaccines against SARS-CoV-2 and how barriers to vaccination can potentially be addressed.

**Methods:**

We conducted semistructured qualitative interviews with 17 unvaccinated adult persons who inject drugs during August and September 2021 at a New York City syringe service program, where approximately three-fourth of participants identified as Latino (55%) or African American (22%). Interviews lasted roughly 20 minutes. The interview guide examined reasons for declining vaccination, participants’ understanding of COVID-19 risks, and how messages could be developed to encourage vaccine uptake among people who inject drugs.

**Results:**

Participants acknowledged that they faced increased risk from SARS-CoV-2 owing to their injection drug use but feared that long-term substance use may have weakened their health, making them especially vulnerable to side effects. Fears of possible side effects, compounded by widespread medical mistrust and questions about the overall value of vaccination contributed to marked ambivalence among our sample. The desire to protect children and older family members emerged as key potential facilitators of vaccination.

**Conclusions:**

Community-developed messages are needed in outreach efforts to explain the importance of vaccination, including the far greater dangers of COVID-19 compared to possible unintended side effects. Messages that emphasize vaccines’ ability to prevent inadvertently infecting loved ones, may help increase uptake. Community-focused messaging strategies, such as those used to increase HIV and hepatitis C virus testing and overdose prevention among people who inject drugs, may prove similarly effective.

## Introduction

People who inject drugs face drastically increased risk of exposure to SARS-CoV-2, the virus that causes COVID-19, and are far more likely to experience severe complications, including death, if they develop COVID-19 [1]. Because COVID-19 principally targets the lungs, it can be especially harmful to individuals who experience respiratory toxicity due to opioids [2,3] (this includes overdose-related respiratory depression) [3] and presents additional risk to polysubstance users who inject opioids and smoke tobacco, cannabis, crack cocaine, or other drugs.

People who inject drugs are frequently homeless [4] or living on streets or in unhygienic settings that severely limit their access to clean water and handwashing [5]. Many also experience crowded, poorly ventilated conditions in shelters and group living settings that make social distancing impossible [5]. The close personal contact required to obtain drugs introduces additional risks of SARS-CoV-2 infection [6], which further increases if people share drugs or equipment. Moreover, to reduce the risk of overdose, People who inject drugs are frequently advised not to inject alone because if something went wrong, no one would be around to help them. Unfortunately, while this prevents overdose deaths, it can cause additional COVID-19 risk.

At the same time, owing to stigma [7,8], lack of access to health care [5], and widespread medical mistrust [9] rooted in a history of discrimination and systemic racism, many people who inject drugs, who are most at risk, are highly unlikely to get vaccinated. Prior research indicates that before the current pandemic, people who inject drugs frequently avoided testing and treatment for hepatitis C virus (HCV) because they feared inappropriate or disrespectful treatment from medical staff [9]. Likewise, although hepatitis B virus (HBV) infections are frequently attributed to injection drug use, people who inject drugs are less likely to vaccinate against HBV compared to those who do not inject drugs [10], and among persons who inject drugs who do vaccinate against HBV, two-thirds of individuals do not complete the 3-dose vaccination series [11]. A recent study shows nearly half of individuals with substance use disorders (SUDs) are unwilling to vaccinate against COVID-19 [12], underscoring the critical need for new messaging and enhanced outreach to this population.

For African American and Latino people who inject drugs, the disproportionate impact of COVID-19 can be especially deadly [1,5]. COVID-19 has been concentrated in African American and Latino communities nationwide [13,14] since the start of the pandemic, and the effects are clearly visible in New York City [15-17], where this study was conducted. In parts of the South Bronx and East Harlem—New York City areas with mostly African American and Latino populations—COVID-19 deaths per 100,000 people reached roughly 10 times those in majority White areas of Manhattan [16]. Nonetheless, in a survey published in late 2020, fewer than half of African American respondents and fewer than two-thirds of Latino respondents indicated that they would receive COVID-19 vaccines if they were available free of charge [18]. In a 2021 study, 60% of White respondents said that they would get vaccinated against COVID-19, but only 36% of African Americans indicated that they would [19]. A 2021 nationwide study of vaccination rates by race, ethnicity, and state found that although numbers are improving among underrepresented groups, relative uptake rates of COVID-19 vaccination among White adults were higher by a median factor of 1.3 times compared to African American adults and by a median of 1.3 times compared to Latino adults as well, highlighting the need to promote vaccine equity [20].

There is a rich history of messaging guided by evidence-based theories of behavior change (eg, Social Cognitive Theory [21] and the Information, Motivation, Behavioral Skills Model [22]). This includes studies examining how messages can be tailored for, and evaluated among, specific groups (eg, underserved populations who attend church [23]; African American and Latino adolescents [24]). In the course of multiple National Institutes of Health–funded studies designed to encourage health behavior change among underserved populations, our team has developed a methodology focused on community members’ expertise. We first conduct in-depth qualitative interviews to understand barriers to, and facilitators of, specific health behaviors among members of a selected population, and then iteratively develop technology-based interventions in response. We have used this methodology to increase HIV testing among emergency department patients [25,26], to increase HIV and HCV testing and overdose prevention among people who inject drugs [27], and to encourage people who inject drugs to carry naloxone kits to reverse overdose events [28]. Accordingly, to inform technology-based intervention messaging aimed at encouraging African American and Latino people who inject drugs to vaccinate against SARS-CoV-2, our team conducted qualitative interviews to examine why people who inject drugs in low-income, historically disadvantaged communities in New York City choose not to get vaccinated. This study describes our initial findings.

## Methods

### Overview

A purposive sample of unvaccinated people who inject drugs (n=17) was recruited in August and September 2021 from individuals receiving services at a community-based syringe service program (SSP) with a participant base of mostly African American and Latino people who inject drugs in New York City. Staff from the SSP recruited participants in a drop-in-center at SSP headquarters. Participants were recruited from the population of people who come to the organization for a host of services on a weekly or daily basis. Eligibility criteria for interviewees included injection drug use within the past 90 days, and not having received a vaccination against COVID-19, both assessed via self-report. Once it was determined they were eligible, they were referred to members of our research team who explained details of the study. To ensure both anonymity and to prevent duplicate responses, we used program participants’ existing SSP anonymous identifiers as their study identification. After participants provided verbal informed consent, 1 of 2 project staff members—who are coauthors of the current paper—conducted interviews on site in private rooms at the SSP. Interviews lasted approximately 15-30 minutes and were audio-recorded and transcribed verbatim by a third-party service. Participants received US $20 cash at the end of the interview as compensation for their time.

Interviews were conducted in English and Spanish by project staff, using an interview guide developed by the principal investigator (PI) with input from the full project team, including members of the partnering organization. Preliminary interview topics were developed from an exhaustive literature review on vaccine hesitancy and COVID-19. Two members of the study team who were experienced qualitative interviewers and researchers conducted semistructured interviews that explored both barriers to and facilitators of COVID-19 vaccination including the following: COVID-19 knowledge, sources of information, perceptions of vaccine risk, vaccine uptake, vaccination settings, stigma, medical mistrust, and policy-related barriers to and facilitators of vaccination. As part of the discussion of potential facilitators, interviewers asked participants to suggest messages our team could share with other people who inject drugs to increase vaccination rates. The study team also collected demographics from study participants, including race and ethnicity, gender, and primary language spoken at home.

Transcripts were analyzed through thematic analysis. Following completion of each audio-recorded interview, the digital file was promptly transcribed by external transcription services: REV.com for English language interviews and Datagain Services for interviews conducted in Spanish. The PI made sure interview files did not contain any identifying data before submitting them for transcription. Coding was conducted using MAXQDA qualitative analysis software.

Our team met weekly to conduct preliminary analysis of the interview transcripts to identify broad thematic categories addressed in each interview and to discuss these identified thematic categories which consisted of both a priori constructs (based on the aims of the study and the interview guide) and emerging themes (that were related to the study aims but not specifically anticipated). An initial code list was developed from the interview guide and project aims. To expand this preliminary code list, a small subset of interviews was jointly analyzed by 3 team members, and codes and coding strategies were then discussed by the larger team.

Three team members, including 2 who also conducted interviews, used an inductive or deductive approach to analyzing the interviews using a combination of a priori and emergent code categories to identify some of the barriers to and facilitators of COVID-19 vaccination [29]. Additional codes were added during discussions with community partners at the SSP. Codes were compared for thematic consistency and discrepancies were processed during regular team meetings to ensure intercoder reliability. Two authors then independently coded the remaining 15 transcripts, frequently meeting with the study team after coding sets of five interviews to discuss and resolve any coding discrepancies by consensus.

To characterize the range of reasons for declining a vaccination and the most salient ones, we calculated the frequency with which themes were endorsed by study participants. This is not to imply that our qualitative data can be generalized to larger populations of people who inject drugs or to vaccine decliners, but to better characterize the range of themes that emerged among a population of people who inject drugs in New York City. As other qualitative studies have suggested, infrequently endorsed themes can be as important for designing public health interventions as frequently endorsed ones [30]. Once we reached a point where no new thematic areas were identified in newly conducted interviews, we determined by consensus that saturation had been reached; at this point, we completed scheduled interviews and stopped recruiting new interviewees. This process resulted in a total of 17 interviews.

### Ethical Considerations

All procedures, including the interview guide, were approved by a single governing institutional review board, BRANY (protocol number 21-039-524), and the study participants provided informed consent.

## Results

### Participant Characteristics

Seventeen people who reported injection drug use in the past 90 days and had not been vaccinated were interviewed for this study. Participants identified as male (n=11, 64.7%) and female (n=6, 35.3%). The majority of participants identified as Hispanic or Latino (n=11, 64.7%), 5 (29.4%) identified as Black non-Hispanic, and 1 (5.9%) identified as White non-Hispanic. A chief goal of our research was to keep interviews brief and focused on why participants declined vaccination and how potential barriers to vaccination could be addressed. An additional important goal was to protect the privacy of respondents. As a result, we did not include questions about participants’ backgrounds beyond self-reported race and gender.

### Barriers to Vaccination

Participants described multiple reasons for not getting vaccinated, ranging from a fear of side effects (both known and as of yet undiscovered), to misinformation and medical mistrust, as well as questions about whether the vaccines were even needed. Participants also reported understanding they faced increased risk of SARS-CoV-2 exposure because of their injection drug use, but said they feared long-term drug use could have weakened their immunity and overall physical condition, rendering them especially vulnerable to negative vaccine side effects. Details of related findings, accompanied by specific quotes illustrating each emerging theme, appear below.

#### Side Effects (Endorsed by 17 of 17 Interviewees)

This fear of side effects included not only immediate reactions, but also reports of people who took the vaccine and within days or weeks developed other health problems or died. These fears appear compounded by concerns the vaccine was released only a short time after the first cases of COVID-19 were reported: “six months later, we got a vaccine. How did they do that?” [Interviewee 6, Hispanic man]. Accordingly, a number of participants reported a desire to wait and see if additional negative effects would emerge over time.

I don't want to get it, and then, you know, three months from now, all of a sudden, um, people who are 40 and under are dying because they, they're, something with the vaccine, how it interacts with our, our brain chemicals as we reach the age of 40. … I've been waiting to see. You know? I'd say five years would be a good period of time to get all the ducks in a row, to know if it's worth it or not.

Interviewee 14, non-Hispanic White man

I'm like wait a minute … there's a chance I'm taking. I know it's a- it's a better thing for me, like just do it and get it out the way and- and hope not to catch the virus. But it's another thing for me to think “Wait a minute, but if I do it what will happen to me?” I'm not sure I might get a side effect, a bad side effect, some- you know, detrimental to my life or whatever.

Interviewee 7, Hispanic man

I literally seen somebody caught a reaction next to me on the bus, just came off from getting the Johnson & Johnson. They never made it out of the bus without the paramedics. So, um... I don't know. I want to, but I don't want to. I don't know how it's gonna look in the future. I don't know how the future will be. I was gonna... I was just gonna put that to the future, but... I'm pretty sure they're gonna come up with something better, with vaccines.

Interviewee 6, Hispanic man

As highlighted by the last quote, many participants claimed firsthand knowledge of someone who became ill after vaccination. However, it remains unclear how many of these incidents are attributable to the vaccine, and which might still have happened (eg, a person getting sick on a bus) even if the person had not vaccinated.

#### Mistrust (Endorsed by 9 of 17 Interviewees)

Interview data indicate that other theories are circulating and spread by social media and television, as well as by close friends and family members, and they have created strong barriers to vaccination.

You see the TV. You have Farrakhan, Minister Farrakhan. “Don't take this vaccination. It killed this one, it killed that one.” … “Oh, the government is putting a chip inside you” and so on and so on.

Interviewee 15, Non-Hispanic Black man

Lotta people don't vaccinate because they say, “Oh,” 'scuse my language, “Oh, that's bullshit,” you know. 'Scuse me for expression. You know, “That's, that's, it's a lie or it's the fake. It's just the government, you know, trying to, another way of keeping track of you,” you know, and stuff like that.

Interviewee 3, Hispanic man

#### Barriers Specific to People who Inject Drugs (Endorsed by 5 of 17 Interviewees)

Two additional themes that emerged through our interviews appear specific to people who inject drugs. Some participants indicated that maintaining a relatively healthy lifestyle, combined with steps they take to protect themselves from HIV—such as only using new syringes and not sharing injection equipment—would protect them from COVID-19 just as well as vaccination:

I've been doing good with my own regimen. You know, washing my hands, eating right, so on and so on. I've been doing good so far and I figure I don't need it.

Interviewee 15, Non-Hispanic Black man

Other participants recognized that the close contact of social interactions related to drug use made them increasingly vulnerable to COVID-19 and therefore required additional safeguards. At the same time, some within our sample questioned whether the toll that years or decades of drug use had taken on their physical health made them even more susceptible to potential vaccine side effects.

Especially as drug users … it attacks us first. Because we come in contact with all different kind of people and they be high and they don't take care they life … They don't take, medicate, nothing … So we are high risk. So therefore as, as us being high risk, we should be at the door, knocking at the door like bang, bang, bang, let me in.

Interviewee 13, Non-Hispanic Black woman

Well I've been a drug user for a lot of years, number of years, and I don't know if my body will be able to take- I'm not- I'm not sure about catching certain side effects that can be detrimental to my life. You know? … I'm scared of that fact that I don't know what to expect. I mean I know I have a better chance at surviving from getting at if I am vaccinated. However … if I do take the vaccination I'm not sure what might happen to me. You know? I'm not sure if I might get a side effect … or die. Die from it because of my drug use over the years.

Interviewee 7, Hispanic man

### Facilitators

Interviews with our sample of unvaccinated people who inject drugs also established a number of potential vaccination facilitators. Participants spoke of wanting to protect family members from inadvertent exposure, especially children who were too young to receive vaccination and older adults who, in general, faced greater risk from COVID-19. Participants also reported that simply discussing the increased risk of COVID-19 exposure among substance users might facilitate vaccination uptake, if handled correctly. Interview data underscore the value of phrasing intervention messages in positive terms (eg, “getting vaccinated will help you because…”), and the importance of not making intervention recipients felt as though vaccination is being forced upon them.

#### Protecting Family and Members of One’s Social Network (Endorsed by 5 of 17 Interviewees)

Children, mothers, and grandmothers all emerged as potential facilitators of vaccination among our sample. As noted earlier, participants expressed a strong desire to protect their families. In addition, participants noted that mothers could prove to be excellent messengers for and models of vaccination uptake.

I would tell them to do it, that they should think about their children, they wouldn't want that to happen to their children, or anybody else.

Interviewee 1, Hispanic man

If my mother tells me. She has told me so many times too. She is vaccinated too. My mom. Thank God. And she is fine. … And my grandma. Thank God, they are already fine, and they are vaccinated.

Interviewee 16, Hispanic man

#### Importance of Vaccination for People who Inject Drugs (Endorsed by 9 of 17 Interviewees)

Similar to concerns about vaccination among people who inject drugs, which emerged among our sample, themes about messages that would facilitate vaccination among people who inject drugs emerged as well. These included messaging about the increased dangers of injection drug use during the pandemic, which again underscore the risks stemming from the close personal contact associated with injection drug use:

I recommend vaccination to any person using drugs on the streets. They should be vaccinated, because they are on the streets, and they don't know who they may infect if they are infected. You don't know … When you use drugs, you interact with many people, and you may infect many people. So, I would tell them to get vaccinated as soon as possible. That's what I say.

Interviewee 17 Hispanic man

#### Importance of Positive Message Tone (Endorsed by 17 of 17 Interviewees)

Suggested methodologies to increase vaccination among people who inject drugs also included simple reassurance as a facilitator, including telling people that they would not be left alone in their time of need.

Something like that, “If you're afraid, I'll go with you.” Yeah … just to encourage them by saying that it does- I, I, it doesn't hurt, you know. Only take a couple of seconds, and, and it's over. And you're good.

Interviewee 9, Hispanic man

Lastly, although our sample only included people who had not vaccinated against COVID-19, some participants indicated that the process of discussing their resistance to vaccination during study interviews and describing how they might explain the importance of vaccination to someone else left them more likely to accept a vaccination if one were offered.

I would say, “Listen ... with me, myself, I never got the vaccine.” I will try 'cause, you know, really, I, I'm scared, but ... I'm sitting here with you, so that's why I'm try- I would like to get the vaccine.

Interviewee 11, Hispanic woman

## Discussion

### Principal Findings

In many ways, our sample may be described as more “vaccine ambivalent” than “vaccine hesitant” or “vaccine resistant.” Interview data show participants are clearly aware of their increased risk of COVID-19 exposure, and that participants understand the importance of vaccination to prevent illness. At the same time, participants expressed concerns about the vaccines’ safety, and whether a possible weakening of their bodies owing to injection drug use has intensified the danger of possible vaccine side effects. In addition, participants in our sample, like others in our society, describe a seemingly endless stream of social media misinformation, often repeated by close friends and family members, which actively discourages vaccination. Together, these issues have left our participants unsure what steps they should take to protect themselves and what sources of information they can trust.

This ambivalence becomes more important to address as the danger of COVID-19, specifically among substance users, is increasingly well documented. According to a large, nationwide study conducted by the National Institutes of Health and researchers at Case Western Reserve University [31], risk of hospitalization and death from COVID-19 is strongest among those recently diagnosed with opioid use disorder (OUD), which includes heroin injectors. Further, among individuals recently diagnosed with OUD, risks are significantly stronger among African American people who use opioids than among White people who use opioids (risk among Hispanic or Latino people who use opioids was not specifically analyzed by the study) [31]. Thus, given the composition of our sample in which the majority of participants were by far African American or Latino, it would be logical to expect that an understanding of these risks would in itself encourage vaccination. Nonetheless, interview data indicate that barriers to vaccination described above, and in particular those related to potential side effects, strongly discouraged participants from getting vaccinating even when they clearly understood their increased risk from COVID-19 and expressed a desire to protect themselves and people close to them.

Participants repeatedly mentioned that they feared their history of drug use would exacerbate the dangers of potential side effects, possibly to the point that vaccination might prove deadly. These concerns may be particularly difficult to dispel among people who inject drugs, who are contemplating vaccination and, in fact, appear to have scientific merit. Clinical trials for the approved COVID-19 vaccines did not explicitly include participants with SUD, and there are no current systematic studies examining the real-world effectiveness of COVID-19 vaccines among SUD or populations of people who inject drugs [32]. Additionally, a large nationwide analysis published in October 2021 indicates that fully vaccinated people who were diagnosed with SUD are at significantly higher risk for breakthrough infection than fully vaccinated people who have not been diagnosed with SUD [32]. Thus, although it is completely understandable when an interviewee says they want to wait 5 years to see if vaccination is worthwhile and safe, curtailing the spread of COVID-19 among people who use drugs and others at the highest risk of infection requires far more immediate action.

Some interviews suggest participants became more amenable to vaccination during their discussions with our team. This may indicate that simply allowing interviewees to voice their concerns about vaccination without being judged or silenced encouraged participants to consider the benefits of vaccination and left them feeling empowered to make positive decisions about protecting themselves. A similarly supportive and noncoercive approach could potentially encourage vaccination—and health care utilization in general—on a wider scale among people who inject drugs and other members of marginalized, high-risk populations, especially if paired with messaging designed to address known barriers to vaccination and emphasize facilitators (eg, “No matter what you do we want you to be safe, and the best way to keep yourself, your kids, and people you care about safe from COVID is to vaccinate”). This type of strategy, rooted in the idea of low-threshold access to health care via harm reduction services [33,34], could prove particularly valuable if vaccine mandates are shown ineffective among our target population. Not all the people who inject drugs in our sample have jobs; hence, employer mandates might not fully increase vaccination rates. Similarly, many people in our sample might not seek to dine at restaurants or visit museums; hence, related vaccination requirements may similarly have little effect.

### Next Steps

Building upon our team’s participatory methodology, we now plan to create a series of digitally delivered intervention messages based on the aforementioned findings, including short videos and SMS text messages to encourage vaccination among people who inject drugs, who initially decline. All steps of intervention development and evaluation will include continued involvement among people who inject drugs, including ongoing monthly meetings with a community advisory board who we have consulted since August 2021.

### Limitations

This study is not without limitations, the first one related to the generalizability of a fairly small sample recruited at a New York City SSP. Owing to the relative saturation of area harm reduction services, participants may feel less stigma and may therefore be more open to discussing substance use and related health issues, including decisions to decline vaccination. This may also be a strength of our study, as interviews conducted in a supportive SSP environment may have resulted in more detailed, and possibly more honest, participant responses. Additionally, our analyses show the themes discussed in this study emerged among a number of interviews. This suggests current data may represent larger topics of concern among people who inject drugs in the New York City area. Further research is warranted to examine whether similar themes would be present among additional populations of people who inject drugs nationwide.

### Conclusions

Effectively encouraging vaccination among people who inject drugs remains a public health priority. If members of populations of people who inject drugs, who are generally already greatly underserved by health care, do not get vaccinated in adequate numbers, SARS-CoV-2 can continue to spread and mutate into potentially more dangerous variants. Appeals to protect the health of loved ones and the safety of the larger community have been successfully employed to encourage important health behaviors among populations of people who inject drugs, such as HIV or HCV testing and prevention along with overdose prevention and reversal. Developing intervention materials that depict trusted community members (eg, recognizable peer educators and individuals with compelling personal stories) delivering clear, nonjudgmental messages may prove especially helpful now. Similarly, organizations that currently offer services to people who inject drugs, including SSPs or other community-based organizations, can facilitate vaccination by offering easy access to vaccines on site (people may be more likely to accept a vaccine offer from someone they already know and trust, especially if they do not have to travel to an additional location).

Importantly, data from this study show that people who inject drugs often remain reluctant to get vaccinated even when they understand the dangers posed by COVID-19, and the increased safety vaccines can offer to them and their families. Given historic vaccine hesitance and medical mistrust among people who inject drugs, combined with pervasive concern about possible side effects, interventions are urgently needed to effectively offer vaccines in ways those most in need will actually accept them.

## Abbreviations

HBV: hepatitis B virus
HCV: hepatitis C virus
OUD: opioid use disorder
PI: principal investigator
SSP: syringe service program
SUD: substance use disorders
